# Whole-genome sequencing-based phylogeny, antibiotic resistance, and invasive phenotype of *Escherichia coli* strains colonizing the cervix of women in preterm labor

**DOI:** 10.1186/s12866-021-02389-7

**Published:** 2021-12-03

**Authors:** Marvin Williams, Alyssa B. Jones, Amanda L. Maxedon, Jennifer E. Tabakh, Cindy B. McCloskey, David E. Bard, Daniel P. Heruth, Susana Chavez-Bueno

**Affiliations:** 1 Department of Obstetrics and Gynecology, University of Oklahoma Health Sciences Center, 800 Stanton L. Young Blvd, OK 73117 Oklahoma City, USA; 2 University of Missouri Kansas City, 2411 Holmes Street, MO 64108 Kansas City, USA; 3 Division of Infectious Diseases, Children’s Mercy Hospital Kansas City, UMKC School of Medicine, 2401 Gillham Road, 1st floor Annex, 1501.13, MO 64108 Kansas City, USA; 4 Department of Pathology, University of Oklahoma Health Sciences Center, 800 Stanton L. Young Blvd, MO 73117 Kansas City, USA; 5 Developmental and Behavioral Pediatrics, University of Oklahoma Health Sciences Center, 800 Stanton L. Young Blvd, MO 64108 Oklahoma City, USA; 6 The Children’s Mercy Research Institute, Children’s Mercy Kansas City, MO 64108 Kansas City, USA

**Keywords:** *Escherichia coli*, Preterm labor, Neonatal sepsis, Whole genome sequencing, Molecular phylogenetics, Antibiotic resistance

## Abstract

**Background:**

*Escherichia coli* is a major neonatal pathogen and the leading cause of early-onset sepsis in preterm newborns. Maternal *E. coli* strains are transmitted to the newborn causing invasive neonatal disease. However, there is a lack of data regarding the phenotypic and genotypic characterization of *E. coli* strains colonizing pregnant women during labor.

**Methods:**

This prospective study performed at the University of Oklahoma Medical Center (OUHSC) from March 2014 to December 2015, aimed to investigate the colonization rate, and the phylogeny, antibiotic resistance traits, and invasive properties of *E. coli* strains colonizing the cervix of fifty pregnant women diagnosed with preterm labor (PTL). Molecular analyses including bacterial whole-genome sequencing (WGS), were performed to examine phylogenetic relationships among the colonizing strains and compare them with WGS data of representative invasive neonatal *E. coli* isolates. Phenotypic and genotypic antibiotic resistance traits were investigated. The bacteria’s ability to invade epithelial cells *in vitro* was determined.

**Results:**

We recruited fifty women in PTL. Cervical samples yielded *E. coli* in 12 % (n=6). The mean gestational age was 32.5 (SD±3.19) weeks. None delivered an infant with *E. coli* disease. Phenotypic and genotypic antibiotic resistance testing did not overall demonstrate extensive drug resistance traits among the cervical *E. coli* isolates, however, one isolate was multi-drug resistant. The isolates belonged to five different phylogroups, and WGS analyses assigned each to individual multi-locus sequence types. Single nucleotide polymorphism-based comparisons of cervical *E. coli* strains with six representative neonatal *E. coli* bacteremia isolates demonstrated that only half of the cervical *E. coli* isolates were phylogenetically related to these neonatal invasive strains. Moreover, WGS comparisons showed that each cervical *E. coli* isolate had distinct genomic regions that were not shared with neonatal *E. coli* isolates. Cervical and neonatal *E. coli* isolates that were most closely related at the phylogenetic level had similar invasion capacity into intestinal epithelial cells. In contrast, phylogenetically dissimilar cervical *E. coli* strains were the least invasive among all isolates.

**Conclusions:**

This pilot study showed that a minority of women in PTL were colonized in the cervix with *E. coli*, and colonizing strains were not phylogenetically uniformly representative of *E. coli* strains that commonly cause invasive disease in newborns. Larger studies are needed to determine the molecular characteristics of *E. coli* strains colonizing pregnant women associated with an increased risk of neonatal septicemia.

**Supplementary Information:**

The online version contains supplementary material available at 10.1186/s12866-021-02389-7.

## Background

*Escherichia coli* has surpassed group B Streptococcus (GBS) as the most common cause of early-onset sepsis (EOS) in premature newborns. Traditional teaching has held that GBS was responsible for EOS in term newborns; however, recent data demonstrates in selected US areas *E. coli* is the most common pathogen associated with sepsis in term newborns [1]. Rates of *E. coli* EOS continue to rise, particularly in preterm very-low-birth-weight infants, increasing in this population from 10 cases per 1000 births twenty years ago to 14 cases per 1000 births in the last five years with associated mortality up to 40 % [2–4]. Maternal colonization with neonatal pathogens is the primary risk factor for neonatal sepsis [5]. This is well-established for GBS, which ascends from the birth canal and infects the newborn causing septicemia [6]. GBS colonization rates, and the genomic characteristics of GBS strains that colonize pregnant women are well described, but these data are lacking for *E. coli* in pregnancy. Despite the limited characterization of the strains colonizing pregnant women and their potential pathogenic traits, *E. coli* transmission from mother to newborn has been documented [7]. Animal models have also demonstrated that vaginal colonization with *E. coli* close to the time of delivery produces neonatal infection [8]. It is therefore relevant to better understand the rate of *E. coli* colonization in pregnant women, and the characteristics of these strains.

Previous research has demonstrated that *E. coli* is a fecal colonizer in 100 % of pregnant women [9, 10]. However, the prevalence of *E. coli* in the genital tract of pregnant women is not well-defined, particularly in the United States. Moreover, unlike GBS, antibiotic resistance in invasive *E. coli* strains is widespread and has progressively worsened [4]. As the incidence of invasive neonatal *E. coli* continues to increase, it is essential to understand the microbiologic characteristics of maternal strains that newborns are closely exposed to during the perinatal period. Only then can effective interventions be created to optimize treatment approaches against neonatal sepsis.

The objectives of our pilot study were the following (1) Determine the *E. coli* cervical colonization rate in women in preterm labor. We focused on women in preterm labor because this diagnosis increases the risk of delivering a preterm newborn with greater susceptibility to infection [11]. (2) Characterize the strains’ phylogeny, and phenotypic and genotypic antibiotic resistance characteristics. (3) Assess the invasive capacity of the recovered bacteria using an *in vitro* epithelial cell invasion model.

## Results

### Subjects’ clinical characteristics and pregnancy outcomes

We identified 50 women in preterm labor (PTL) who met the criteria for study participation from March 2014 through December 2015. The demographic and clinical characteristics of the subjects are shown in Table 1.

Cervical colonization of *E. coli* was present in six women, for a prevalence of 12 % (95 % CI 2.99-21.01). There was no statistical significance in race distribution, maternal chronological age, gestational age at which the cervical sample was collected upon enrollment, and length of stay between women colonized with *E. coli* compared to noncolonized women. The rate of delivery in both groups was similar. No study participant had a history of urinary tract infection within a month before sample collection or reported a history of fever within the week preceding enrollment in the study.

**Table 1:** Clinical Characteristic of women in preterm labor with or without *E. coli* colonization

	All Subjects	Non-Colonized	Colonized
	50	44 (88)	6 (12)
Race			
• White • Hispanic • African-American • American-Indian • Asian • Other	27 12 5 2 1 3	24 10 5 1 1 3	3 2 0 1 0 0
Median maternal age in years (25 %-75 %)	26.1 (23.3-30.1)	25.2 (22.5-29.9)	27.6 (25.5-32.7)
Mean GA at Sample Collection (±SD)	32.9 (2.91)	32.9 (2.99)	32.8 (2.23)
Median length of hospital stay in days (25 %-75 %)	3 (2-5.25)	3 (2-5.75)	2 (1-10)
Delivered	27 (54)	24 (54.5)	3 (50)
Presence of ROM	13 (26)	13 (29)	0
Chorioamnionitis			
• Yes • No • Unknown	3 (6) 43 (86) 4 (8)	3 (7) 37 (84) 4 (9)	0 6 (100) 0
Nugent classification			
• Normal • Intermediate • Bacterial vaginosis • Invalid	20 (40) 13 (26) 16 (32) 1 (2)	17 (38.6) 12 (27.3) 14 (31.9) 1 (2.2)	3 (50) 1 (16.7) 2 (33.3) 0
GBS screening result			
• Positive • Negative • Not performed	11 (22) 34 (68) 5 (10)	9 (20.5) 30 (68.2) 5 (11.3)	2 (33.3) 4 (66.6) 0

Legend: Data are presented as No. (%) unless otherwise indicated

GA, Gestational age in weeks; GBS, group B *Streptococcus*; ROM, Rupture of membranes; PTL, Pre-term Labor

Compared with non-colonized versus colonized women, rupture of membranes and placental histopathologic chorioamnionitis occurred only in the non-colonized group. No cases of clinical chorioamnionitis were diagnosed. The presence of chorioamnionitis was unknown in four women, all without *E. coli* cervical colonization. Both groups had similar rates of bacterial vaginosis and concurrent GBS colonization. Twenty-seven women were delivered before discharge. Pathology examination was performed in 16 of the 27 delivered placentas; none of these mothers were colonized by *E. coli.* Median gestational age at delivery was 34.6 weeks (IQR 31.3-35.6). All newborns were singleton and survived until hospital discharge at a median age of 8 days of life (IQR 5-47). None of the newborns developed *E. coli* bacteremia in either group

Antibiotics were administered within the first seven days of life to 52 % of newborns born to noncolonized women, whereas each newborn born to the colonized mothers received antibiotics in the same period. The median (25 %-75 %) length of stay in the hospital was 6.5 (3.2-42) days vs. 20 (3.5-33) days for the newborns born to noncolonized vs. colonized women, respectively. These differences were not statistically different likely due to the small sample size

A detailed description of the clinical characteristics of the 50 subjects included in the study can be found in Additional file 1

### Clinical microbiology laboratory data and genotypic antibiotic resistance of *E. coli* cervical isolates

Phenotypic antibiotic susceptibility testing of *E. coli* cervical isolates by the clinical microbiology laboratory showed that most isolates were susceptible to all antibiotics tested by clinical standard methods. One isolate, SCBcol-5, was multi-drug resistant. None of the isolates met the criteria for extensive drug resistance [12] (Table 2). The K1 capsule was present in all isolates.

**Table.2:** Antibiotic susceptibility testing and presence of K1 capsule by agglutination test

*E. coli* Cervical Isolate	Antibiotic Resistance	K1 capsule agglutination test
SCBcol-1^a^	• Ampicillin • Ampicillin/sulbactam • Piperacillin • Tetracycline	Positive
SCBcol-2	None	Positive
SCBcol-3	• Tetracycline	Positive
SCBcol-4	• Tetracycline	Positive
SCBcol-5^a^	• Ampicillin • Ampicillin/sulbactam • Piperacillin • Tetracycline • TMP/SMX	Positive
SCBcol-6	None	Positive

Legend: TMP/SMX, Trimethoprim and sulfamethoxazole

^a^Intermediate to cefazolin and ticarcillin/clavulanic acid

**Fig. 1:**
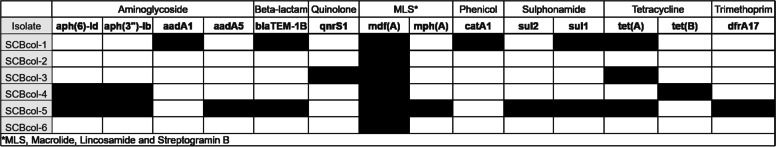
Presence of genes related to antibiotic resistance in cervical *E. coli* isolates from women in preterm labor. Legend: Whole genome sequencing data was analyzed to determine the presence of antibiotic resistance genes using the ResFinder 4.0 database available from the Center from Genomic Epidemiology as described in the Methods section. [13–15]. Shown are only genes that were present in at least one strain (black cells). SCBcol-5, a multi-drug resistant isolate, showed the greatest number of antibiotic resistance genes

WGS data analyses to determine antibiotic resistance genes were consistent with the phenotypic resistance patterns of these isolates as tested by the clinical microbiology laboratory. Accordingly, antibiotic resistance genes were more prevalent in isolates SCBcol-1 and SCBcol-5 than in isolates SCBcol-2 and SCBcol-6 (Fig. 1).

### Phylogenetic relatedness of cervical *E. coli* strains

Quadruplex PCR phylogroup classification demonstrated that cervical *E. coli* isolates were distributed in diverse phylogroups. Two isolates belonged to B2, a phylogroup most commonly associated with neonatal *E. coli* bacteremia in the US and other countries (Table 3) [16–19]. One cervical isolate belonged to phylogroup F, which is related to phylogroup D, another phylogroup represented in isolates causing neonatal disease. Whole-genome sequencing (WGS)-based multi-locus sequence type (MLST) classification revealed that the cervical *E. coli* isolates were heterogeneous, and only one belonged to ST131, a common sequence type (ST) found among neonatal invasive isolates in recent years.

**Table.3:** Phylogroup and MLST classification of cervical *E. coli* isolates colonizing women in preterm labor

Maternal Cervical *E. coli* isolates	Phylogroup	Multi-locus sequence type
SCBcol-1	F	ST-31
SCBcol-2	E	ST-68
SCBcol-3	C	ST-1495
SCBcol-4	B1	ST-4252
SCBcol-5	B2	ST-131
SCBcol-6	B2	ST-491

Legend: Phylogroup assignment was performed by PCR methodology and sequence type assignment was done by data analyses of WGS data as described in the Methods section

The *E. coli* isolates colonizing the cervix of women in PTL in our study were not associated with neonatal invasive disease in their offspring. This finding prompted us to investigate the phylogenetic relationship of this group of cervical strains with representative *E. coli* isolates commonly associated with neonatal bacteremia. Very few studies in the United States have described the genetic characteristics of invasive neonatal *E. coli* in newborns at the sequence type level. Our recent work showed that ST95 and ST131 predominate among neonatal *E. coli* bacteremia isolates [20]. Weissman et al. similarly found ST95 to be the most prevalent ST among invasive neonatal *E. coli* isolates, followed by ST69 [21]. We, therefore, sought to compare the phylogenetic relationship of cervical colonizing *E. coli* strains with neonatal *E. coli* bacteremia strains belonging to ST69 (isolates SCB5 and SCB29), ST95 (SCB12 and RS218), and ST131 (isolates SCB34 and SCB58), which we have characterized in our laboratory [20, 22–24].

**Fig. 2:**
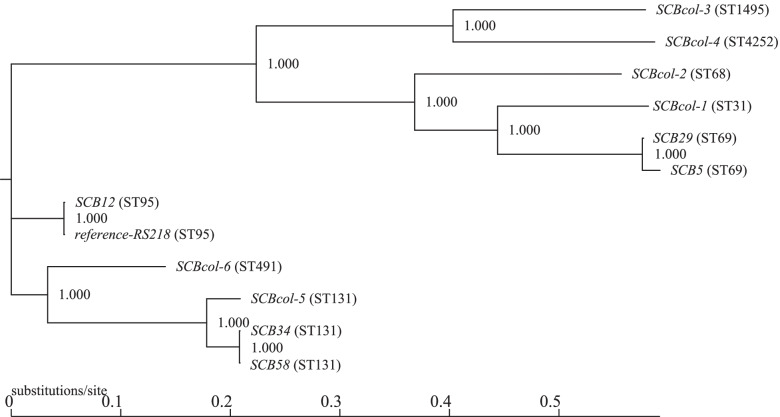
Single nucleotide polymorphism (SNP)-based phylogenetic tree comparing cervical *E. coli* isolates and representative neonatal *E. coli* bacteremia strains. Legend: WGS data was used to perform phylogenetic comparisons of *E. coli* cervical colonization isolates with neonatal bacteremia isolates. Data were analyzed with CSI Phylogeny 1.4 and a tree was constructed to show the relatedness among neonatal and cervical isolates [25] The SNP-based tree shows that the colonizing strain SCBcol-5 was the closest related to the neonatal bacteremia isolates SCB34 and SCB58, all belonging to the same ST131. The least related cervical colonization isolates in relation to neonatal isolates were SCBcol-2, SCBcol-3, and SCBcol-4

The archetypal ST95 neonatal invasive *E. coli* isolate RS218 was used as the reference sequence for constructing the maximum likelihood phylogenetic tree shown in Fig. 2. This analysis showed close relatedness of SCBcol-1 to neonatal isolates SCB5 and SCB29. Not surprisingly, SCBcol-5 (ST131) was closely related to the neonatal isolates belonging to ST131, namely SCB34 and SCB58. SCBcol-6 was most related to neonatal ST95 strains SCB12 and RS218. The colonizing strains least related to neonatal strains were SCBcol-2, SCBcol-3, and SCBcol-4. These results demonstrate that *E. coli* isolates capable of colonizing the cervix of pregnant women are heterogeneous, and as a group, not fully phylogenetically representative of *E. coli* strains commonly causing neonatal invasive disease.

**Fig. 3:**
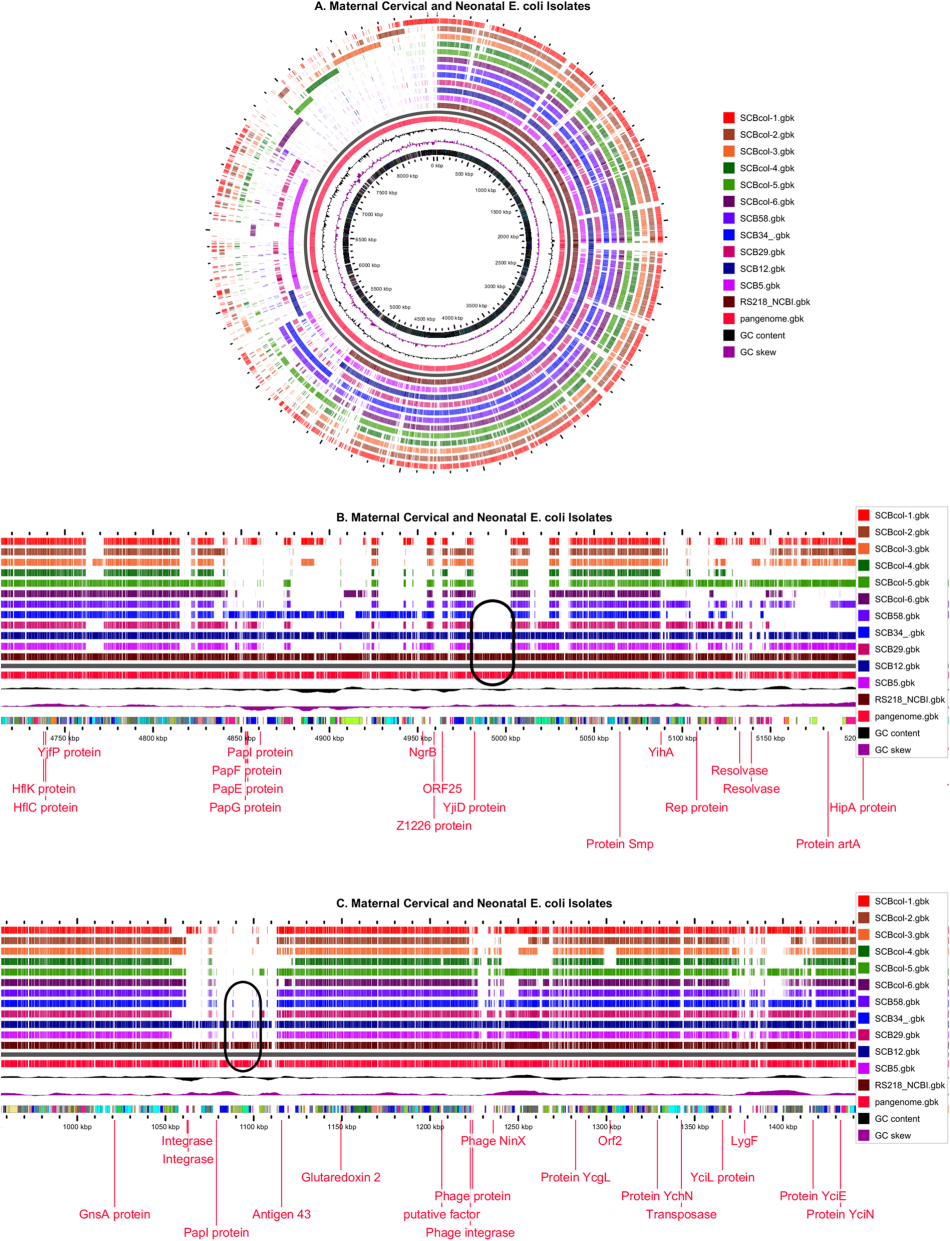
Visualization of pangenome analysis comparing cervical colonizing *E. coli* isolates with representative neonatal *E. coli* invasive strains. Legend: **A** is a circular representation of all isolates included in the analyses. The innermost circle represents the position of all features on the pangenome. Rings 2 and 3 show the GC skew and content, respectively. Ring 4 represents the pangenome, separated by a black ring from the reference RS218 sequence (ring 6), and the sequences of all additional isolates (rings 7-17). **B** **C** are linear representations of the above comparisons. Figure 3B highlights the location of the *ibeA* gene, which is relevant to the pathogenesis of neonatal meningitis [27–29]. **C** shows the presence of the *iroBCDEN* genes relevant to the synthesis and processing of salmochelin, a siderophore involved in the pathogenesis of invasive *E. coli* infections [30]. The location of these specific genes is indicated by a black oval in **B** **C**

To further examine differences in the full complement of genes among cervical and neonatal *E. coli* strains, we performed a pangenome analysis using the Gview server and its genome visualization tool. *E. coli* RS218 was used as the seed genome and the complete genomes of all cervical and neonatal isolates were added to generate the circular representation shown in Fig. 3. This graphic representation is constructed by using the Basic Local Alignment Search Tool (BLAST) to compare the reference sequence with the WGS data of each additional isolate by constructing a reference pangenome, and the results are included on the map [26]. All the sequences, including the RS218 (reference) sequence were uploaded in GenBank format, therefore allowing feature information to be extracted and comparisons generated in a tabular format as well (Additional file 2).

Pangenome comparisons demonstrated that > 50 % of genes present in neonatal invasive isolates were shared with maternal strains, especially those in SCB5 and SCB29. However, there were genes present only in some neonatal strains, and among them, we identified *ibeA* in strains RS218 and SCB12 (Fig. 3B) (Additional file 2). This gene encodes the invasion of brain endothelium A (IbeA) protein that contributes to the development of meningitis by neonatal *E. coli* strains [27–29]. Genes encoding for the siderophore salmochelin, *iroBCDEN*, (Fig. 3 C) were also only present in RS218 and SCB12. The complete list of gene content comparisons among all strains is included in Additional file 2.

Figure 3 also shows several genomic regions specific to individual cervical isolates absent in neonatal isolates and that were shared only with partial regions of other cervical isolates. These regions include genes encoding several hypothetical proteins, fimbrial proteins, secretion system components, and membrane proteins, among others (Additional file 2). Taken together, our findings indicate an overall richer genome diversity of colonizing cervical strains compared to invasive neonatal *E. coli* strains.

### Epithelial invasion by maternal and neonatal *E. coli* strains

**Fig. 4:**
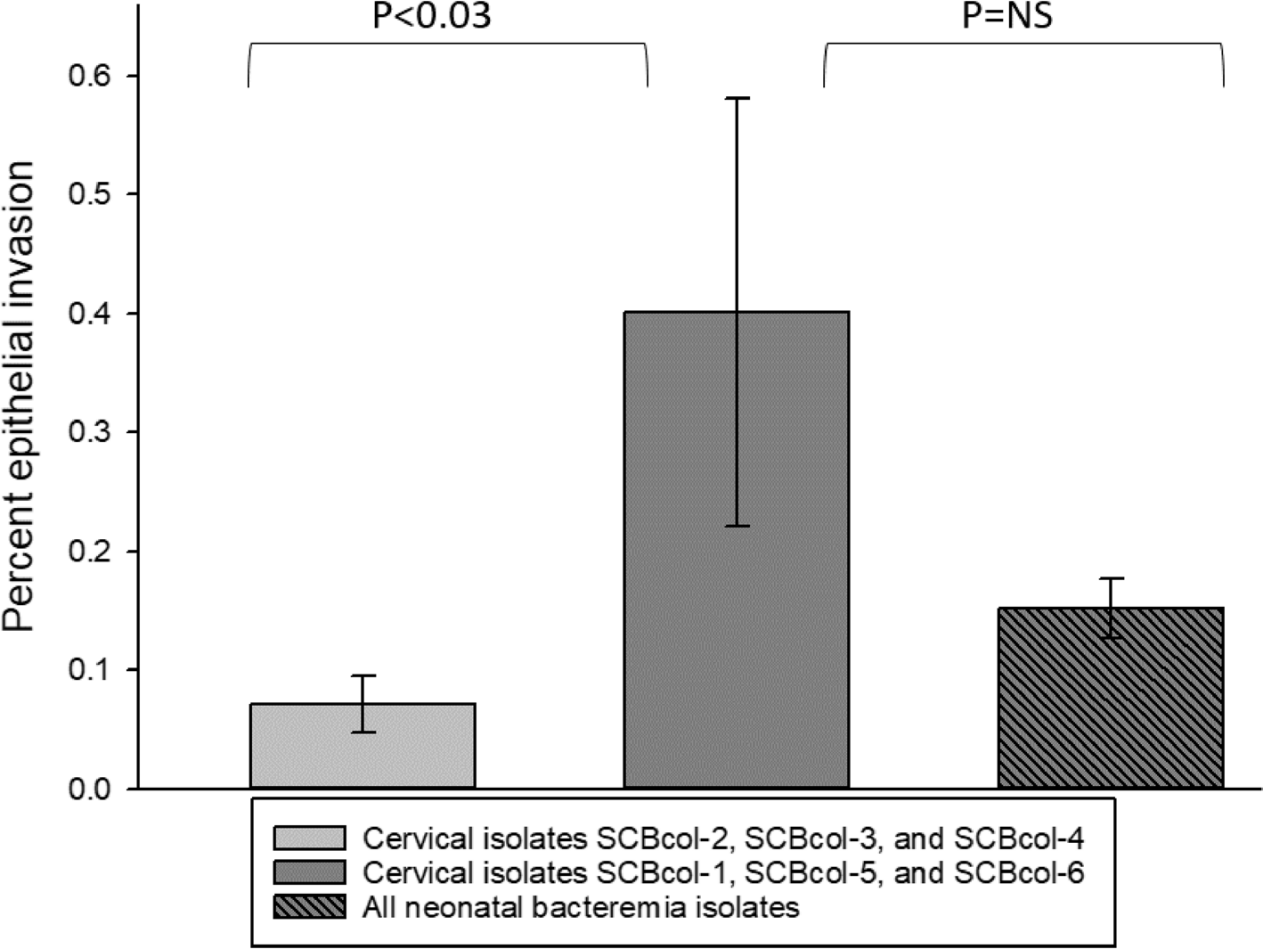
Epithelial invasion comparisons of cervical colonization and invasive neonatal *E. coli* isolates. Legend: The invasion capacity of *E. coli* cervical isolates and neonatal bacteremia isolates was compared using an intestinal epithelial cell invasion model *in-vitro* [23]. Cervical colonization isolates most closely related to neonatal bacteremia isolates at the phylogenetic level (SCBcol-1, SCBcol-5, and SCBcol-6) showed the greatest invasion capacity as a group, as compared to the group of least phylogenetically related colonization isolates (P<0.03, one way ANOVA)

The invasive ability of maternal cervical *E. coli* isolates was assessed using an epithelial invasion *in vitro* model using intestinal T84 cells. The percent invasion of each isolate varied from 0.02 % (SD±0.007) to 1.16 % (SD±0.44) (Additional file 3). Given the phylogenetic similarities of cervical isolates SCBcol-1, SCBcol-5, and SCBcol-6 with the selected neonatal invasive isolates (Fig. 2), we compared their epithelial invasion capacity as a group with the invasion ability of the group of neonatal strains included in the study. As demonstrated in Fig. 4, *in vitro* invasiveness of SCBcol-1, SCBcol-5, and SCBcol-6 was comparable to that of neonatal isolates. On the other hand, the group of cervical isolates that were most distantly phylogenetically related at the phylogenetic level, i.e., SCBcol-2, SCBcol-3, and SCBcol-4, had significantly lower invasion capacity (p<0.03).

## Discussion

The purpose of our pilot study was to investigate the presence of *E. coli* colonizing the cervix of women diagnosed with PTL. Our objective was to better understand the phylogeny, antibiotic resistance traits, and invasive properties of the *E. coli* strains recovered in this relevant clinical scenario. We focused on colonization of the cervix, the highest anatomical site in the genital tract that can be accessed by clinical examination in a non-invasive manner and is the location of colonizing microorganisms with the potential to access the amniotic cavity and infect the newborn.

Our sample cohort demonstrated a prevalence of *E. coli* cervical colonization of 12 % (95 % CI 2.99-21.01). Several studies have reported a variable rate of carriage of *E. coli* in vaginal samples from pregnant women (from 12 % to 19 % in North and South America and Europe). Still, data on cervical colonization are scarce, particularly in recent years [31]. A study in Europe documented a similar prevalence of *E. coli* of 14 % in cervical samples of women in PTL [32]. On the other hand, endocervical colonization rates were lower at 3.5 % among women in labor in Switzerland [33] and higher at 24 % in Iranian women with premature preterm rupture of membranes ROM [34]. Our pilot study provides initial information on the prevalence of *E. coli* cervical colonization in US women in PTL. Our study was descriptive and was not powered to establish whether *E. coli* cervical colonization was a risk for neonatal sepsis in our population. Because of very small sample size, we did not find statistical differences between groups regarding race distribution, maternal chronological age, gestational age, and length of hospital stay between the groups of colonized vs. noncolonized women. Likewise, differences in neonatal clinical outcomes cannot be determined from this study. Additional studies are needed to define the risk of vertical transmission of *E. coli* from colonized mothers to their newborns in relation to various clinical variables, including the possible role that the amount of *E. coli* colonization may play on clinical outcomes. Larger studies will aid to determine the most effective screening strategies in pregnant women that would inform potential preventative measures against neonatal *E. coli* sepsis.

We did not find evidence of widespread multi-drug resistance among the cervical *E. coli* isolates that we recovered in this population of women in PTL. Data on antibiotic resistance of *E. coli* isolates colonizing the genital tract of pregnant women are limited and vary according to geographic location. Widespread resistance to beta-lactam antibiotics in *E. coli* is an increasing concern. Prevalence of extended-spectrum beta-lactamases (ESBL)-producing *E. coli* colonizing pregnant women has been reported between 2 and 7 % in South Asia, Europe, Africa, and Japan [35–39], and as high as 15-40 % in India [10, 40]. It is possible that we did not find extensively resistant or ESBL-producing *E. coli* strains in our study because we excluded pregnant women with a recent history of antibiotic use, which increases the risk of colonization with resistant organisms [10]. However, one of the cervical isolates, which belonged to ST131 demonstrated MDR. This clonal group is frequently associated to antibiotic resistance and can be found in asymptomatic pregnant women [41]. Larger studies are needed to determine the rates of resistance in *E. coli* colonizing pregnant women over time more accurately. Our study included detailed characterization of the genetic determinants of antibiotic resistance in the colonizing strains we recovered along with corresponding phenotypic resistance data. Our findings are in concordance with previous studies demonstrating that genotypic susceptibility testing can be as reliable as phenotypic testing [13]. Ongoing surveillance of antibiotic resistance in *E. coli* strains that colonize pregnant women is crucial, as this may impact treatment decisions for antimicrobial therapy. While antibiotic resistance in neonatal *E. coli* invasive is not widespread in the US yet, worrisome trends have already emerged that warrant close monitoring [42, 43].

The phylogeny of *E. coli* isolates colonizing the genital tract of pregnant women has not been explored in detail. Our phylogenetic analyses showed that half of the cervical strains belonged to phylogroups typically associated with severe invasive *E. coli* infections, including neonatal sepsis, i.e., phylogroup B2, and the related phylogroup F [44, 45]. We also demonstrated colonization with strains classified in phylogroups B1, C and E, which more commonly behave as commensals [46]. Other studies from Spain and Japan have reported a prevalence of phylogroup B2 of 60 %-70 % among vaginal and endocervical *E. coli* isolates from pregnant women [47, 48]. In Africa, however, vaginal isolates from pregnant women belonged predominantly to the traditionally less pathogenic phylogenetic groups A and E [49]. Possible explanations of these differences may include the diverse anatomic locations for sample collection, geographic factors, and the pregnancy stage and obstetric complications of subjects included in the various studies.

Our study included MLST, a more refined method to study *E. coli* phylogeny [50]. MLST analyses showed that the colonizing isolate SCBcol-5 belonged to ST-131, an ST commonly found among invasive neonatal *E. coli* isolates in the US. Two other colonizing isolates were also phylogenetically similar to *E. coli* STs associated with invasive infections in newborns [20, 21]. Moreover, we demonstrated that these related cervical isolates had a significantly greater ability to invade epithelial cells than the more phylogenetically distant cervical strains. Despite the observed phylogenetic and phenotypic characteristics, none of the cervical isolates were associated with invasive disease in the offspring of the colonized mothers in our study. This is most likely related to the overall low rates of neonatal sepsis in the United States. Other possible explanations include gestational age beyond the threshold of extreme prematurity, and the lack of other risk factors such as premature rupture of membranes or chorioamnionitis. Another possible explanation is the lack of virulence genes specific to the pathogenesis of neonatal sepsis in the cervical *E. coli* strains we isolated. As demonstrated by our Gview comparisons, genes known to be relevant to *E. coli* invasive infections such as those encoding IbeA and siderophore-related proteins were only present in neonatal invasive isolates and not in any cervical strains. Interestingly, all the cervical strains showed the presence of the K1 antigen, a virulence factor known to be present in approximately 80 % of *E. coli* isolates causing neonatal meningitis, and about 50 % of *E. coli* strains causing neonatal sepsis [20, 21, 51, 52]. While it has been well established that the K1 capsule facilitates *E. coli* survival in the blood and modulates bacterial trafficking and enhances survival in brain microvascular endothelial cells [53], its role as a risk factor for maternal-child transmission is not defined. The entire repertoire of *E. coli* virulence factors involved in the pathogenesis of neonatal *E. coli* invasive infections has not been defined. As such, it may be possible that among the genes present only in neonatal strains that were isolated, are those important in the pathogenesis of neonatal *E. coli* sepsis (Additional file 2). We also demonstrated that the cervical *E. coli* strains carried genes that were not present in the neonatal invasive strains. We speculate that these genes may confer a better ability to colonize without necessarily causing invasive disease.

The limitations of our study include a very small number of subjects and its single-center setting. However, its prospective design strengthens the results of this pilot study that addresses the scarcity of data on the rates of *E. coli* in the genital tract of pregnant women, particularly in the United States. Moreover, we focused on women in PTL, a population with the potential for delivering a preterm newborn at high risk for invasive *E. coli* disease and in whom the significance of maternal *E. coli* colonization needs further definition. Our study also contributes with new data on the molecular characteristics of *E. coli* colonizing pregnant women that determine their phylogeny and influence their phenotypic antibiotic resistance patterns and invasiveness potential.

## Conclusions

This study provides much needed information on relevant characteristics of the cervical *E. coli* strains that colonize pregnant women at risk for delivering a preterm infant in whom the susceptibility to infection is high. The pilot nature of this project with very limited sample size does not allow generalization to larger populations. Additional studies will aim at further defining the prevalence and the characteristics of the *E. coli* strains that colonize the genital tract of pregnant women. This information is essential to design appropriate preventive strategies against neonatal *E. coli* sepsis, which currently do not exist.

## Methods

This was a prospective, pilot study aimed at investigating the *E. coli* colonization rate, and the phylogeny, antibiotic resistance traits, and invasive properties of *E. coli* strains isolated from the cervix of women diagnosed with preterm labor (PTL).

### Patient population

This was a prospective study performed at the University of Oklahoma Medical Center (OUHSC), from March 2014 to December 2015. Women >18 years old diagnosed with preterm labor (PTL) between 23 and <37 weeks of gestation from the last menstrual period were eligible for enrollment. Preterm labor was defined as the presence of regular uterine contractions and documented cervical effacement and/or dilatation of at least 2 cm in patients at <37 weeks of gestation [54]. Exclusion criteria included antibiotic therapy within 2 weeks prior to enrollment, history of genitourinary abnormalities, history of pelvic surgery or cervical cerclage, use of tocolytics or steroids during the current pregnancy, and human immunodeficiency virus infection. Demographic and clinical data were collected, including pregnancy complications, and neonatal outcomes. Data were collected for pregnant women through discharge, newborns’ charts were reviewed through discharge and for up to three months after birth for any return visits to our medical system. Cases of chorioamnionitis were assigned if included in the physicians’ diagnoses list at any point during the admission, or if confirmed by placental histopathologic examination. Chorioamnionitis was classified as unknown if data were not collected from the medical record. The obstetricians providing standard of care to the women included in the study were not made aware of whether a woman was carrying *E. coli* once results became available.

### Cervical sample collection

The obstetric standard of care for women in PTL at OUHSC includes a vaginal/cervical examination using a sterile speculum to obtain microbiology samples, and to evaluate for possible cervical dilatation, and to ascertain whether rupture of amniotic membranes has occurred [55]. For our study, two additional swab samples were collected at the time of the routine speculum examination on the day of hospital admission. One sterile swab was used to sample the cervix (avoiding the cervical canal) to test for *E. coli*, and a second swab was used to sample the mid-third of the vagina to prepare a slide for determination of bacterial vaginosis (BV) using a formal scoring system assigned by a pathologist (CBM). The swabs were kept at 4 °C until transported to the clinical laboratory, which occurred within 2 h of collection.

### Clinical laboratory processing

Cervical samples were inoculated onto blood and MacConkey agar plates and into MacConkey broth for enrichment of Gram-negative organisms. Broth was plated to a MacConkey agar plate after 24 h incubation. *E. coli* colonies were selected based on growth/lactose fermentation on MacConkey, indole positivity, and oxidase negativity. *E. coli* identification was confirmed using the MicroScan Negative Urine Combo 61 (NUC61) panel on the MicroScan WalkAway *plus* System (Siemens Corporation). Phenotypic antibiotic susceptibility testing was determined using both the MicroScan NUC61 panel, which included determination of extended spectrum beta lactamase-producing isolates, and the TREK Sensititre GN4F Gram negative MIC Plate (TREK Diagnostic Systems/Thermo Scientific). Antibiotics used for susceptibility testing are listed in Additional File 4. The isolates were also tested for the presence of the K1 capsule with a card latex agglutination test (Wellcogen, Remel Europe Ltd, Dartford, Kent, UK). A single colony of an overnight plate culture of each isolate was tested with the appropriate positive and negative controls according to the manufacturer’s protocol.

The vaginal swab was used to prepare a smear on a glass slide that was heat fixed and Gram stained for determination of BV using the Nugent score [56]. BV scoring was determined by a pathologist (CBM) who was blinded to other study results. This method classified the samples into four categories: Normal, intermediate, bacterial vaginosis, and indeterminate.

### Bacterial genotypic analyses for antibiotic susceptibility testing and phylogenetic relatedness

Bacterial genomic DNA from each *E. coli* isolate was purified with the Qiagen® DNeasy extraction kit. Phylogroup classification was performed with the Clermont quadruplex polymerase chain reaction method using the primers and conditions described earlier [20]. WGS was performed with Illumina HiSeq technology using 250-bp paired-end libraries with 50X coverage. Reads were assembled *de novo* with the A5 assembler pipeline [57]. Contigs were analyzed using the bioinformatics resources available at the Centre of Genomic Epidemiology (CGE) from the Technical University of Denmark (http://www.genomicepidemiology.org/). ResFinder 4.0 was used for genotypic detection of antibiotic resistance including all the available acquired antimicrobial resistance genes in the database. Default threshold for percent identity of 90 %, and minimum length of 60 % were chosen [13, 14]. MLST 2.0 (*E. coli* configuration #1) was used to assign the ST of each strain according to the Achtman scheme [58, 59]. CSI Phylogeny 1.4. from CGE was used to determine the phylogenetic relatedness among the strains based on the concatenated alignment of high quality single nucleotide polymorphisms (SNPs) [25]. The neonatal invasive *E. coli* isolate RS218 was used as the reference genome [24]. Default parameters were used, and a phylogenetic tree was generated selecting the FastTree option. The WGS interactive visualization tool GView was used to perform comparative genome analyses of all isolates [26, 60]. A pangenome reference was created using RS218 as the seed genome and sequentially adding all genomes of cervical and neonatal *E. coli* isolates. This pangenome was then used to compare each individual *E. coli* genome to the pangenome using a BLAST atlas. Default settings on Gview were used, including an identity percent cutoff of 80 %. Circular representations were generated and tabular results of BLASTn comparisons were downloaded in .xls format.

### Comparisons of invasion capabilities among maternal cervical colonizing *E. coli* and neonatal invasive *E. coli* isolates

A modified gentamicin protection assay was used to determine the *E. coli* isolates’ ability to invade intestinal epithelial cells as described before [23]. Briefly, T84 intestinal epithelial cell monolayers were infected with each *E. coli* strain at a multiplicity of infection of 10. Bacteria were allowed to invade for 1 h before washing, followed by the addition of amikacin at a concentration of 200 µg/mL (Sigma-Aldrich; St. Louis, MO) for two additional hours to kill extracellular bacteria. All isolates were verified to be susceptible to amikacin with a minimum inhibitory concentration of ≤ 16 µg/mL. After antibiotic treatment, the T84 cells were washed, lysed with 0.1 % Triton X-100, and the recovered intracellular bacteria were quantified. The percent invasion was calculated as follows: (CFU recovered/CFU inoculated) x 100. Each isolate was tested in triplicate and experiments were repeated two to four times. Mean invasion percent was compared among the strains using one-way ANOVA comparisons; a p value <0.05 was considered significant.

### Statistical methods

Descriptive statistics were used to analyze parametric and nonparametric data, as appropriate. The Fisher exact test was used to compare proportions, and the Student’s t-test or ANOVA were used to compare continuous data.

## Supplementary Information

**Additional file 1.** Clinical characteristics of 50 women diagnosed with preterm labor enrolled in the present study.

**Additional file 2.** BLAST hits table of pangenome analysis that included all cervical and neonatal genome sequences.

**Additional file 3.** Invasion into intestinal epithelial cells by each *E. coli* isolate.

**Additional file 4.** Antibiotics used for susceptibly testing of *E. coli* isolates by the clinical microbiology laboratory.

## Data Availability

The whole-genome sequencing datasets generated and analyzed for each *E. coli* isolate included in the current study were deposited at the National Center for Biotechnology Information (NCBI) with the following GenBank accession numbers. Data will be made available upon manuscript publication. Accession number SCBcol-1 JAHTMT000000000 SCBcol-2 JAHTMU000000000 SCBcol-3 JAHUTW000000000 SCBcol-4 JAHUTX000000000 SCBcol-5 JAHUTY000000000 SCBcol-6 JAHUTZ000000000 SCB5 JAHUUA000000000 SCB12 JMQO00000000 SCB29 JAHUUB000000000 SCB34 JMKH00000000 SCB58 JAHUUC000000000 RS218 JWZW00000000.1.
