# Correction to: Whole-genome sequencing-based phylogeny, antibiotic resistance, and invasive phenotype of *Escherichia coli* strains colonizing the cervix of women in preterm labor

**DOI:** 10.1186/s12866-022-02448-7

**Published:** 2022-02-03

**Authors:** Marvin Williams, Alyssa B. Jones, Amanda L. Maxedon, Jennifer E. Tabakh, Cindy B. McCloskey, David E. Bard, Daniel P. Heruth, Susana Chavez-Bueno

**Affiliations:** 1 Department of Obstetrics and Gynecology, University of Oklahoma Health Sciences Center, 800 Stanton L. Young Blvd, Oklahoma City, OK 73117 USA; 2 University of Missouri Kansas City, 2411 Holmes Street, Kansas City, MO 64108 USA; 3 Division of Infectious Diseases, Children’s Mercy Hospital Kansas City, UMKC School of Medicine, 2401 Gillham Road, 1st floor Annex, 1501.13, Kansas City, MO 64108 USA; 4 Department of Pathology, University of Oklahoma Health Sciences Center, 800 Stanton L. Young Blvd, Kansas City, MO 73117 USA; 5 Developmental and Behavioral Pediatrics, University of Oklahoma Health Sciences Center, 800 Stanton L. Young Blvd, Oklahoma City, MO 64108 USA; 6 The Children’s Mercy Research Institute, Children’s Mercy Kansas City, Kansas City, MO 64108 USA

**Correction to: BMC Microbiol 21, 330 (2021)**

**https://doi.org/10.1186/s12866-021-02389-7**

Following the publication of the original paper [1], the authors spotted a publisher error in “Availability of data and materials” section. The accession number for RS218 needs to be changed to JWZW00000000.1. Corrected section is shown below.

## Availability of data and materials

The whole-genome sequencing datasets generated and analyzed for each *E. coli* isolate included in the current study were deposited at the National Center for Biotechnology Information (NCBI) with the following GenBank accession numbers. Data will be made available upon manuscript publication.

Accession number

SCBcol-1 JAHTMT000000000

SCBcol-2 JAHTMU000000000

SCBcol-3 JAHUTW000000000

SCBcol-4 JAHUTX000000000

SCBcol-5 JAHUTY000000000

SCBcol-6 JAHUTZ000000000

SCB5 JAHUUA000000000

SCB12 JMQO00000000

SCB29 JAHUUB000000000

SCB34 JMKH00000000

SCB58 JAHUUC000000000

RS218 JWZW00000000.1

The original article has been corrected.
