# Supplementary material for: Whole-genome sequencing-based phylogeny, antibiotic resistance, and invasive phenotype of Escherichia coli strains colonizing the cervix of women in preterm labor
Source: BMC Microbiol. 2021 Dec 3;21:330. doi: 10.1186/s12866-021-02389-7 (PMC8641181; doi:10.1186/s12866-021-02389-7)
Supplement: Supplementary file 3 — Additional file 3. Invasion into intestinal epithelial cells by each E. coli isolate. [file 12866_2021_2389_MOESM3_ESM.docx]

| *E. coli* isolates | Median percent invasion (25%-75%) |
| --- | --- |
| **Cervical colonization** |  |
| SCBcol-1 | 0.204 (0.07-0.30) |
| SCBcol-2 | 0.039 (0.035-0.22) |
| SCBcol-3 | 0.025 (0.02-0.03) |
| SCBcol-4 | 0.08 (0.03-0.1) |
| SCBcol-5 | 1.161 (0.85-1.47) |
| SCBcol-6 | 0.056 (0.04-0.07) |
| **Neonatal bacteremia** |  |
| SCB5 | 0.235 (0.10-0.37) |
| SCB12 | 0.067 (0.06-0.07) |
| SCB29 | 0.047 (0.02-0.13 |
| SCB34 | 0.11 (0.05-0.27) |
| SCB58 | 0.16 (0.10-0.22) |
| RS218 | 0.235 (0.08-0.41) |

**Additional file 3.** Invasion capacity into intestinal epithelial cells by cervical and neonatal bacteremia *E. coli* strains.

Legend: Isolates were tested using an in vitro model of invasion using T84 intestinal epithelial cells. Each isolate was tested in triplicate and experiments were repeated two to four times for each individual isolate.
