# Supplementary material for: Whole-genome sequencing-based phylogeny, antibiotic resistance, and invasive phenotype of Escherichia coli strains colonizing the cervix of women in preterm labor
Source: BMC Microbiol. 2021 Dec 3;21:330. doi: 10.1186/s12866-021-02389-7 (PMC8641181; doi:10.1186/s12866-021-02389-7)
Supplement: Supplementary file 4 — Additional file 4. Antibiotics used for susceptibly testing of E. coli isolates by the clinical microbiology laboratory. [file 12866_2021_2389_MOESM4_ESM.docx]

| Amikacin | Ertapenam |
| --- | --- |
| Ampicillin | Gentamicin |
| Ampicillin/sulbactam | Imipenem |
| Aztreonam | Levofloxacin |
| Cefazolin | Meropenem |
| Cefepime | Nitrofurantoin |
| Cefoxitin | Piperacillin |
| Ceftazidime | Piperacillin/tazobactam |
| Ceftriaxone | Ticarcillin/clavulanic acid |
| Cefuroxime | Tigecycline |
| Ciprofloxacin | Tobramycin |
| Doripenem | Trimepthoprim/Sulfamethoxazole |

**Additional File.** Antibiotics used for phenotypic susceptibly testing of *E. coli* isolates.

Legend: Each isolate was tested using standard methodology by the clinical microbiology laboratory at OUHSC. Interpretation of susceptibility testing was made using clinical breakpoint guidelines established by the Clinical and Laboratory Standards Institute (CLSI).
